# Supplementary material for: Humans can infer social preferences from decision speed alone
Source: PLoS Biol. 2024 Jun 20;22(6):e3002686. doi: 10.1371/journal.pbio.3002686 (PMC11189591; doi:10.1371/journal.pbio.3002686)
Supplement: S1 Text — (DOCX) [file pbio.3002686.s001.docx]

**S1 Text. Trial selection prior to running the main experiment.**

In order to maximize the likelihood of observers learning the dictators’ social preferences trial-by-trial, we carefully selected which of the 165 trials would be displayed to the participants. To this end, we considered the trials which would be most informative, in terms of both choices and RT. We ran a linear regression on the RT with the subjective value distance as the independent variable (see **Results** section, **S1A Fig**):

$$RT=b_{0}+b_{1}*|s\left( \mathrm{left} \right)-s\left( \mathrm{right} \right)|$$

*(4)*

We categorized the trials into slow trials, fast trials, and uninformative trials (**S1B Fig**), using a ‘single-peaked’ utility model (**Equation 1**):

$$s\left( \mathrm{left} \right)={1-\left( \mathrm{left}-\mathrm{Pref} \right)}^{2}$$

*(1)*

where $\mathrm{left}$ is the objective value of the left (resp. right) option (i.e., the number of points allocated to ‘self’) and $\mathrm{Pref}$ is the fitted social preference. This single-peaked model assumes that (i) the social preference is unique for each dictator and (ii) between any two allocations that are both on the left (resp. on the right) of the preference, the allocation that is closer to the preference is strictly preferred over the allocation that is further. The specificities of the single-peaked model make it better-suited to explain our Dictator Game data than more complex models such as Fehr-Schmidt or Charness-Rabin model^[[1]](#footnote-1)^. For two choice options $\mathrm{left}$ and $\mathrm{right}$, the single-peaked model gives us the subjective values (or utilities) $s(left)$ and $s(right)$, whose difference in absolute values $D$ predicts the RT:

$$D=|s\left( \mathrm{left} \right)-s\left( \mathrm{right} \right)|$$

*(16)*

And with **Equation 4**:

$$RT=b_{0}+b_{1}*D$$

*(17)*

The coefficients $b_{0}$ and $b_{1}$ respectively represent the intercept and the slope of the model, with $b_{0}$ being around the average empirical RT for the 16 dictators and $b_{1}$ being negative. Now we can invert this linear model to infer $D$ by observing RT:

$$D=\frac{RT-b_{0}}{b_{1}}$$

*(18)*

Because $b_{1}$ is negative, $D\leq0$ when ${RT\geq b}_{0}$. As $D$ is a distance, this implies that as soon as ${RT\geq b}_{0}$, the best prediction is that $D=0$, or $s\left( \mathrm{left} \right)=s\left( \mathrm{right} \right)$ (see **Equation 16**), which means that the predicted preference lies exactly between $\mathrm{left}$ and $\mathrm{right}$. Of course, this is just a single-trial inference, but still the best inference we can make given our model. So, these are the slow decisions we want to look at, those in which:

$${RT_{\mathrm{slow}}\geq b}_{0}$$

*(19)*

For fast decisions, we look at trials in which we can infer that the preference lies outside $\mathrm{left}$ and $\mathrm{right}$. In other words, we want to know whether the RT-inferred utility difference $D_{RT}$ is larger than the utility difference given $\mathrm{Pref}=\mathrm{left}$ or $\mathrm{Pref}=\mathrm{right}$. In this case, by combining $\mathrm{Pref}=\mathrm{left}$ with **Equation 16** and **Equation 1**, we have:

$$D_{\mathrm{Pref}=\mathrm{left}}=\left( \mathrm{right}-\mathrm{left} \right)^{2}$$

*(20)*

Similarly, with $\mathrm{Pref}=\mathrm{right}$, we obtain:

$$D_{\mathrm{Pref}=\mathrm{right}}=\left( \mathrm{left}-\mathrm{right} \right)^{2}$$

*(21)*

Therefore, in the case of fast decisions, **Equation 18** gives us:

$$RT_{\mathrm{fast}}>b_{0}+b_{1}*(\mathrm{left}-\mathrm{right})^{2}$$

*(22)*

In other words, to infer that the preference lies outside $\mathrm{left}$ and $\mathrm{right}$, the RT has to be smaller than $b_{0}$ minus the $b_{1}$-weighted squared difference between $\mathrm{left}$ and $\mathrm{right}$ (**S1 Fig**).

1. For instance, consider the classic Fehr-Schmidt model where $s\left( X_{\mathrm{self}} \right)=X_{\mathrm{self}}-\alpha(X_{\mathrm{self}}-X_{\mathrm{other}})$, with parameter $0<\alpha<1$ representing inequality aversion. For $\alpha=0.5$, all options have the same subjective value. For $\alpha<0.5$, the more an option is unequal, the higher its subjective value. For $\alpha>0.5$, the equal-split option (50/50) will always be preferred. This model would categorize agents with no gradation for the value of $\alpha$. [↑](#footnote-ref-1)
