# Supplementary material for: Humans can infer social preferences from decision speed alone
Source: PLoS Biol. 2024 Jun 20;22(6):e3002686. doi: 10.1371/journal.pbio.3002686 (PMC11189591; doi:10.1371/journal.pbio.3002686)
Supplement: S2 Text — (DOCX) [file pbio.3002686.s002.docx]

**S2 Text. Observers own preference impact their uniformed guesses.**

In the preregistration, we hypothesized that the observers’ own preference might impact their first estimation (before observing anything) and their estimations in the ‘none’ condition. Therefore, we compared their social preference extracted from the Dictator Game task with their reported estimations. We found significant correlations between the observers’ own preference and their first estimation (before any observation; Spearman’s *ρ*(44)=0.38, *p*=.0099, **S2A Fig**) and their average estimation, depending on the amount of information provided to them (average estimation per condition; none: Spearman’s *ρ*(44)=0.56, *p*<.0001; RT only: Spearman’s *ρ*(44)=0.48, *p*=.0018; choice only: Spearman’s *ρ*(44)=0.31, *p*=.038; both: Spearman’s *ρ*(44)=0.27, *p*=.073, **S2B Fig**).
