# Supplementary material for: Humans can infer social preferences from decision speed alone
Source: PLoS Biol. 2024 Jun 20;22(6):e3002686. doi: 10.1371/journal.pbio.3002686 (PMC11189591; doi:10.1371/journal.pbio.3002686)
Supplement: S3 Text — (DOCX) [file pbio.3002686.s003.docx]

**S3 Text. Qualitative model comparison favors the RL model.**

Our full model space included four models: two RL models and two BO models.

In the version of the RL model presented in the main text (**S6A Fig**), the outcome was computed as a weighted sum between the choice-related information and the RT-related information, with the addition of a weight parameter $0<\omega<1$:

$$O_{t}=O_{ch,t}*\left( 1-\omega\right)+ O_{rt,t}*\omega$$

*(12)*

To validate the addition of this free parameter, we tested a simpler version of this RL model where the preference is updated equally from choice-related information and RT-related information. This model was unable to match observers’ behavior in the ‘RT’ condition, where only RT were visible (**S6B Fig**).

We tested two versions of the BO model: the one presented in the main text (**S6C Fig**) included informative priors, which we chose to be Beta(3.5,3) for the estimated preference $0<EP<1$, Gamma(1.2,5) for the temperature $0<\beta<100$, Gamma(2,2) for the boundary separation $0.1<a<10.1$, Normal(0,5) for the drift rate $0<\nu<20$, and a uniform distribution for the non-decision time $0.1<T_{er}<0.5$. For comparison only, we tested a model with uninformative priors, i.e., uniform distributions for all parameters (**S6D Fig**). Both models fail to match observers’ accuracy in the ‘RT’ condition.
