# Supplementary material for: Humans can infer social preferences from decision speed alone
Source: PLoS Biol. 2024 Jun 20;22(6):e3002686. doi: 10.1371/journal.pbio.3002686 (PMC11189591; doi:10.1371/journal.pbio.3002686)
Supplement: S4 Text — (DOCX) [file pbio.3002686.s004.docx]

**S4 Text. Time perception and Social Value Orientation score.**

After the main experiment, observers performed a time perception task. In this part of the experiment, they were shown two successive stimuli, both black squared centered on the screen, for two different time intervals. Observers were asked to report if the second one was displayed for a shorter or longer amount of time than the first one. Each was be composed of a first fixation screen (250ms), a first square shown for some time interval (250ms-2750ms), an interstimulus interval (500ms), a second fixation screen (250ms), a second square shown for a shorter or longer time interval, the decision screen (self-paced), and the intertrial interval (1000ms). The difference in time period between the two stimuli was either 250ms, 500ms, or 750ms for each trial. In the preregistration, we hypothesized that the ability of the observers to discriminate time interval would correlate with their accuracy in the estimation phase, but this was not the case (time perception vs. average accuracy: Spearman’s *ρ*(44)=0.14, *p*=.34; time perception vs. ‘RT’ condition: Spearman’s *ρ*(44)=-0.01, *p*=.93). Accuracy in the Time Perception task are presented in **S10A Fig**.

In the very beginning and at the very end of the experiment, observers were asked to fill in the Social Value Orientation Scale. Results are presented in **S10B Fig**.
