# Supplementary material for: Humans can infer social preferences from decision speed alone
Source: PLoS Biol. 2024 Jun 20;22(6):e3002686. doi: 10.1371/journal.pbio.3002686 (PMC11189591; doi:10.1371/journal.pbio.3002686)
Supplement: S5 Text — (DOCX) [file pbio.3002686.s005.docx]

**S5 Text. Prediction phase’s GLMM sanity check.**

After performing the regression on observers’ ‘prediction’ response times (RT) during their predictions for the dictators, we performed the same regression on observers’ ‘self’ RT, expecting to find no effect of choice visibility nor RT visibility (as this information was only gained for ‘prediction’ responses, after the ‘self’ responses had been made). We ran Generalized Linear Mixed Models (GLMMs) on the winsorized RT (0.05^th^ percentile), with a Gamma distribution of the response variable and an Identity link function, with duration ($\mathrm{duration}$: fast or slow), choice visibility ($\mathrm{infoCh}$: displayed or not; only when predicting other) and RT information ($\mathrm{infoRT}$: displayed or not; only when predicting other) as within-participant factors:

$$RT \sim duration * infoCh * infoRT + \left( 1+duration +infoCh +infoRT \right| observers)$$

As expected, running the GLMM with all factors and interactions only indicated a significant main effect of duration (**S1 Table**). Moreover, removing choice visibility and RT visibility from the GLMM did not significantly impair its goodness-of-fit (*χ^2^*(13)=20.40, *p*=.086, **Fig 3D**, left).
