# Supplementary figures and images for: Humans can infer social preferences from decision speed alone
Source: PLoS Biol. 2024 Jun 20;22(6):e3002686. doi: 10.1371/journal.pbio.3002686 (PMC11189591; doi:10.1371/journal.pbio.3002686)

A

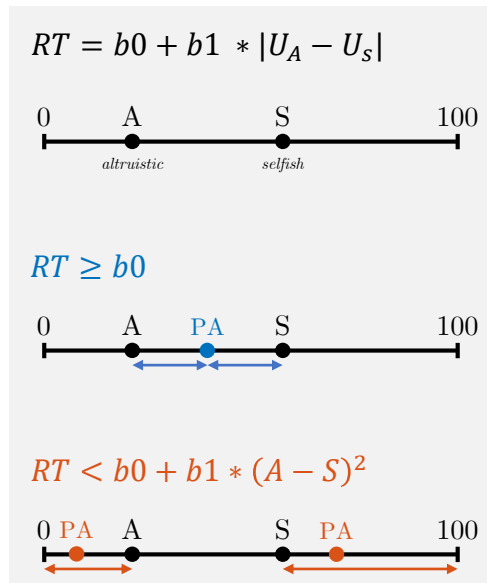

Trial selection

B

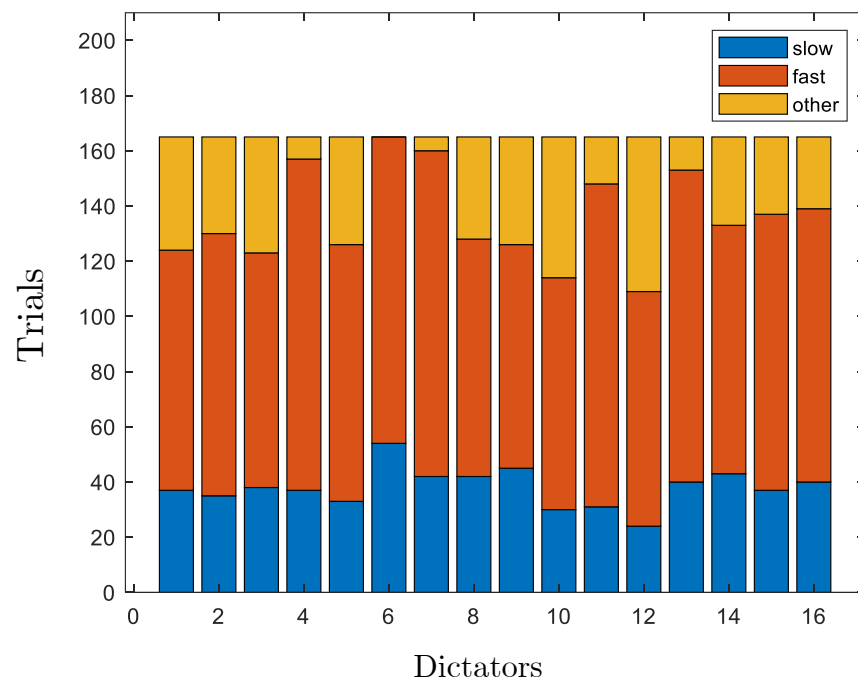

C

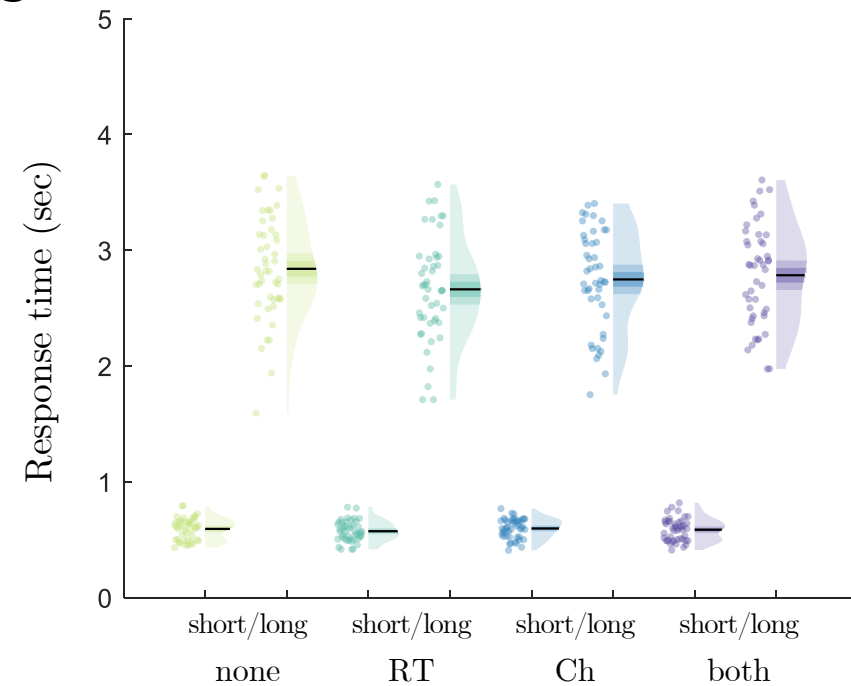

Supplement: S1 Fig — (A) Illustration of the single-peaked model regression with 2 choice options A and S, with A>S without loss of generality. (B) Proportions of all 165 trials performed by the dictators in the Dictator Game experiment, categorized using the single-peaked model. (C) Dictators’ average RT in the selected 6 fast trials (“short RT”) and 6 slow trials (“long RT”), as seen by the observers in each of the conditions. Points indicate individual average, shaded areas indicate probability density function, 95% confidence interval, and SEM. N = 46. Data and analysis scripts underlying this figure are available at https://github.com/sophiebavard/beyond-choices. (PDF) [file pbio.3002686.s006.pdf]

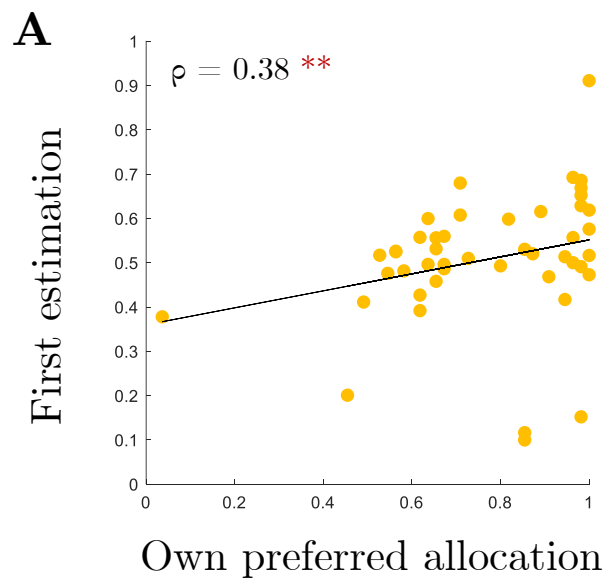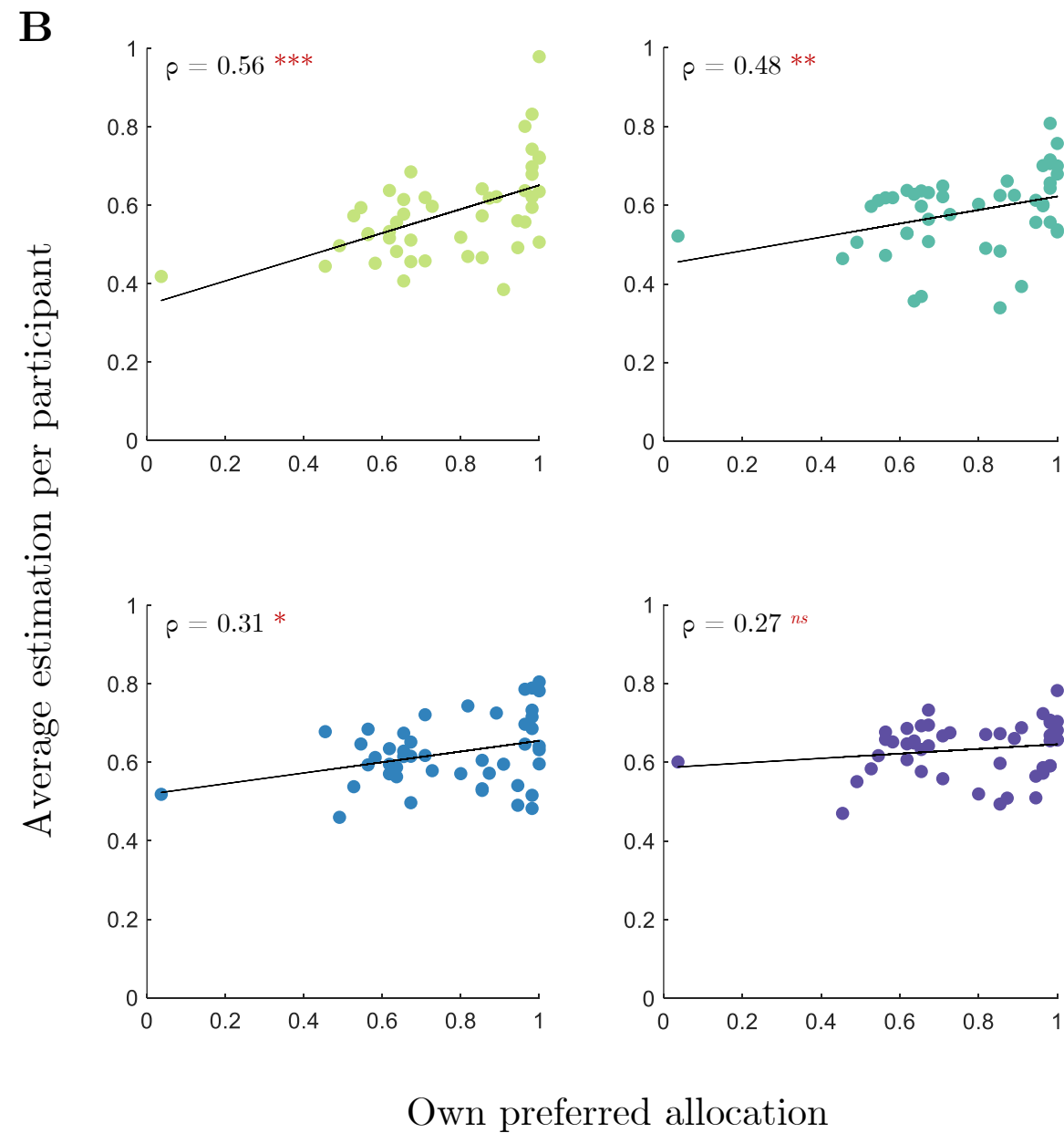

Supplement: S2 Fig — (A) Observers’ average first estimation as a function of their own social preference. N = 46. (B) Observers’ average estimation per condition as a function of their own social preference. ρ: Spearman’s coefficient. N = 48. In all panels, ns: p > 0.05, *p < 0.05, **p < 0.01, ***p < 0.001. Data and analysis scripts underlying this figure are available at https://github.com/sophiebavard/beyond-choices. (PDF) [file pbio.3002686.s007.pdf]

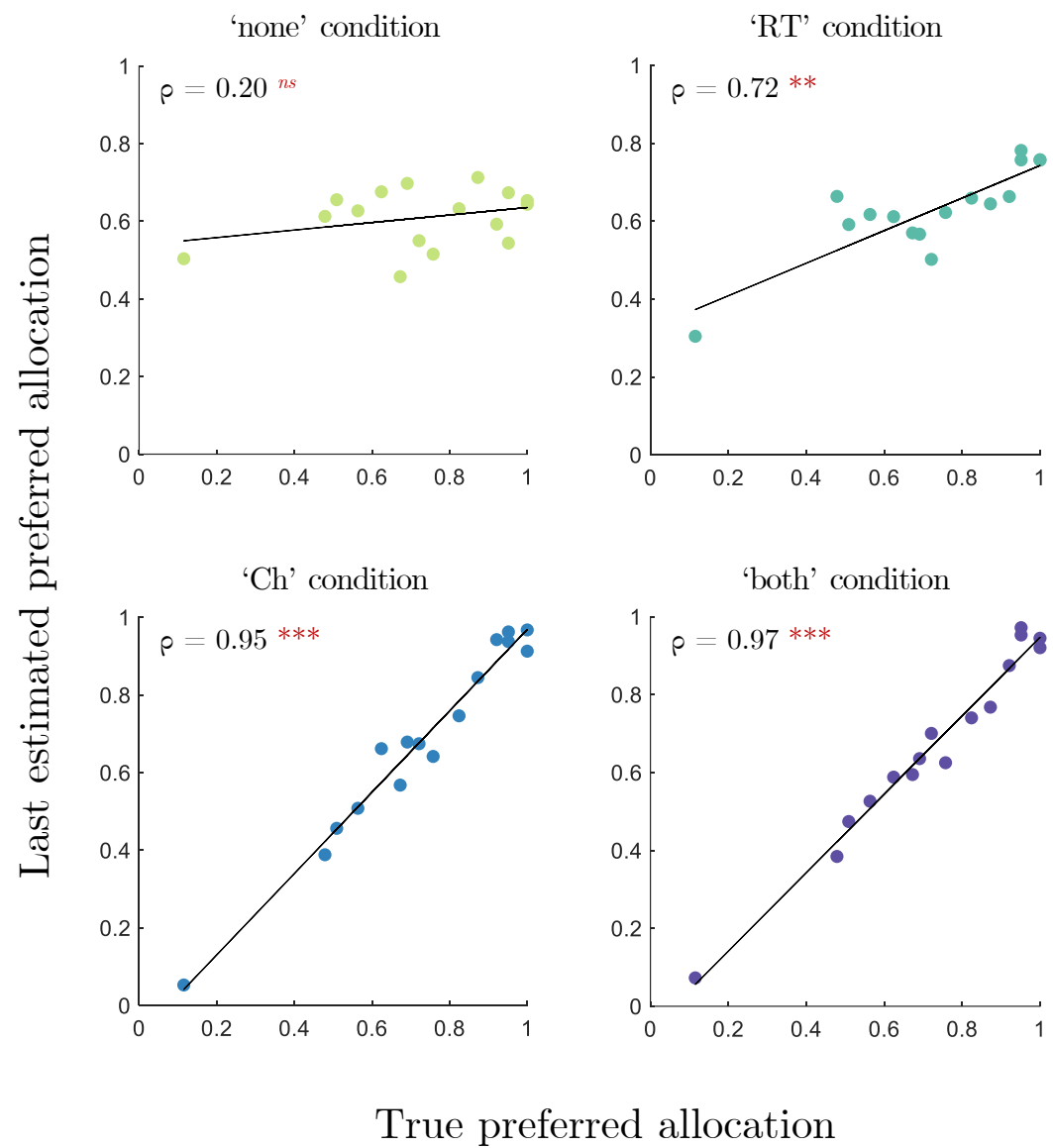

Supplement: S3 Fig — Reported fourth and last estimation per dictator, averaged over observers, as a function of the true preference of each dictator, for each condition. ρ: Spearman’s coefficient. N = 16. In all panels, ns: p > 0.05, **p < 0.01, ***p < 0.001. Data and analysis scripts underlying this figure are available at https://github.com/sophiebavard/beyond-choices. (PDF) [file pbio.3002686.s008.pdf]

**A**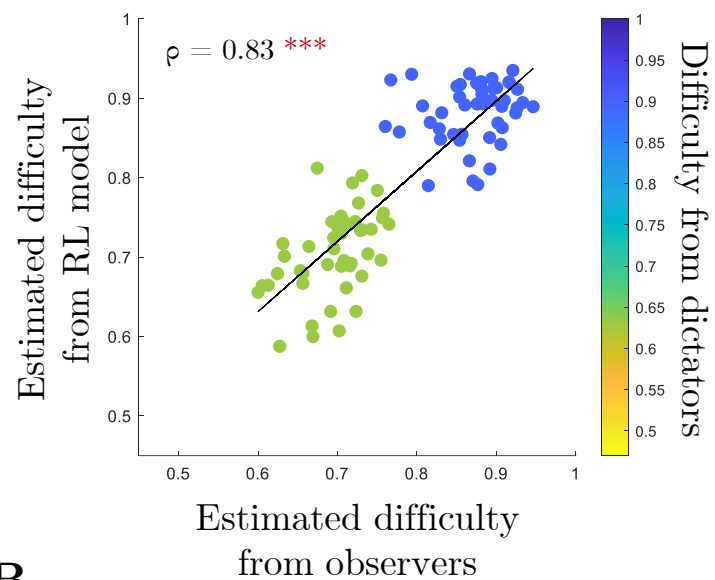**B**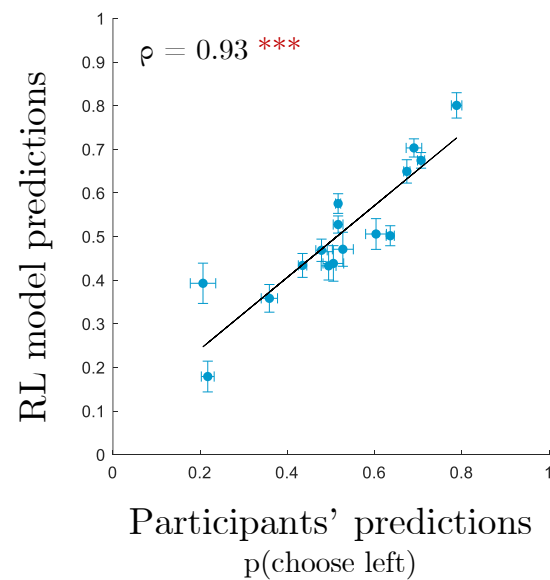**C**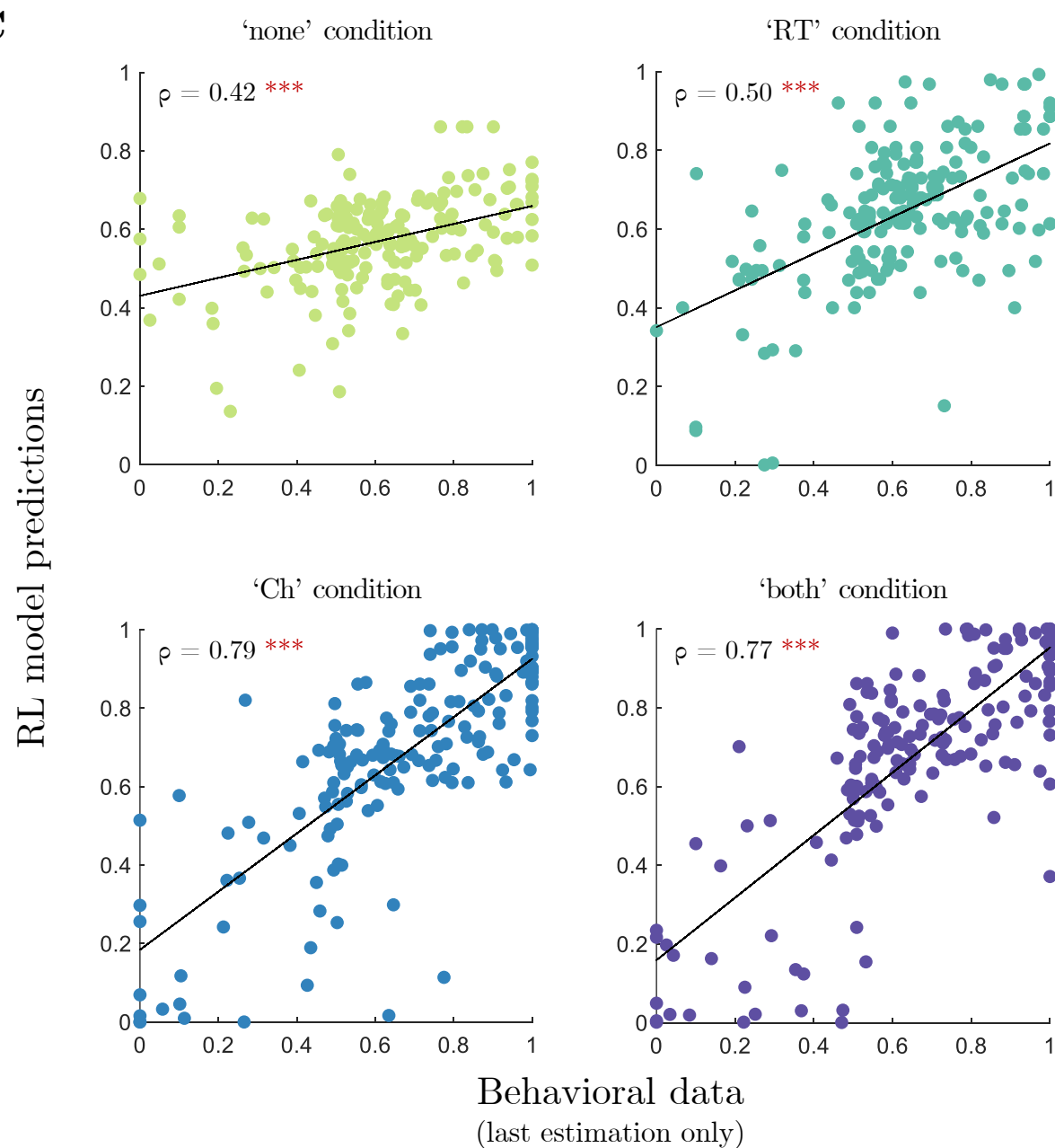

Supplement: S4 Fig — (A) Estimated difficulty extracted from RL model predictions as a function of the estimated difficulty from behavioral data from observers and dictators, after the estimation phase, for trials from the prediction phase. Each point represents one average trial difficulty for each duration (fast/slow) for each observer, averaged over dictators and conditions. N = 92. (B) RL model predictions for the proportion of choices towards the left option in the prediction phase, as a function of the behavioral data. (C) RL model predictions for the fourth and last estimation per observer per observed dictator, as a function of the reported fourth and last estimation, for each condition. N = 184. ρ: Spearman’s coefficient. In all panels, ***p < 0.001. Data and analysis scripts underlying this figure are available at https://github.com/sophiebavard/beyond-choices. (PDF) [file pbio.3002686.s009.pdf]

**A**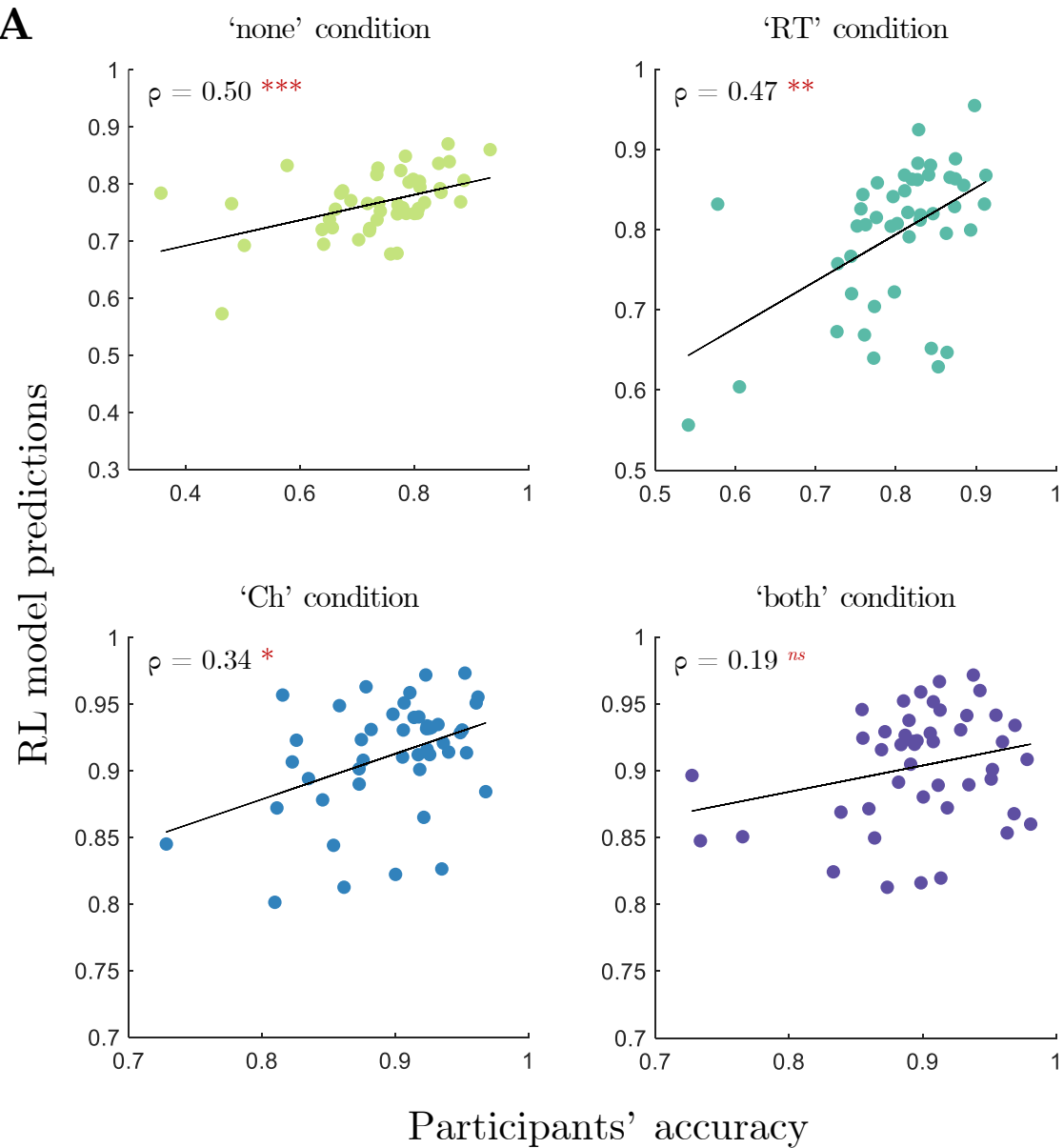**B**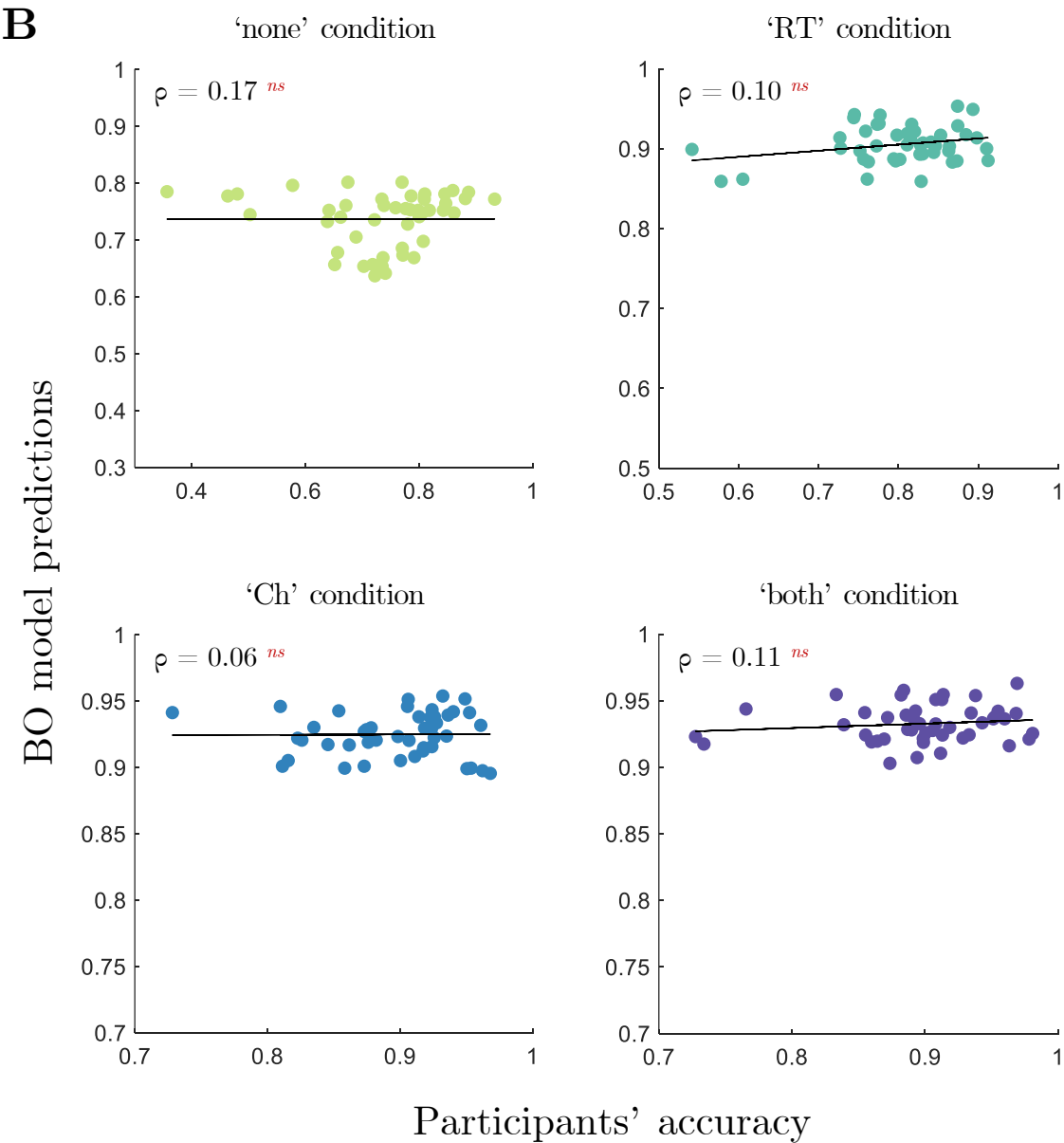

Supplement: S5 Fig — Average accuracy for the last estimation predicted by the RL model (A) and BO model (B) for each condition, averaged over trials and dictators, as a function of the observers’ behavioral accuracy. In all panels, N = 46, ns: p > 0.05, *p < 0.05, **p < 0.01, ***p < 0.001. Data and analysis scripts underlying this figure are available at https://github.com/sophiebavard/beyond-choices. (PDF) [file pbio.3002686.s010.pdf]

**A**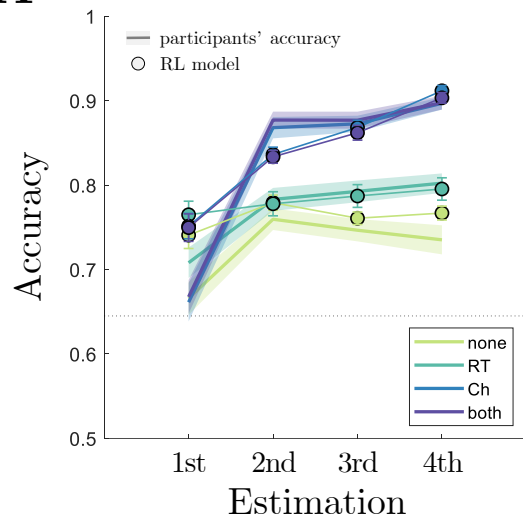**B**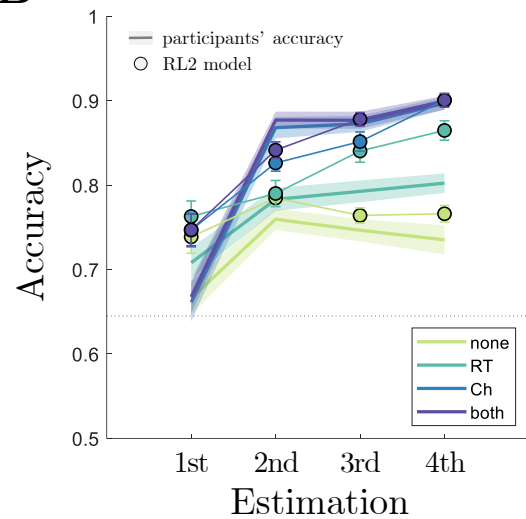**C**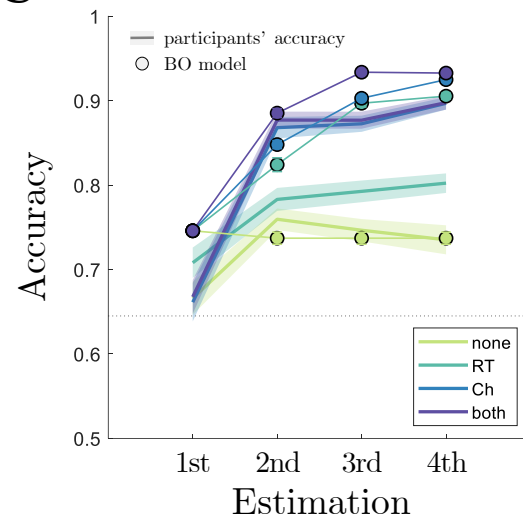**D**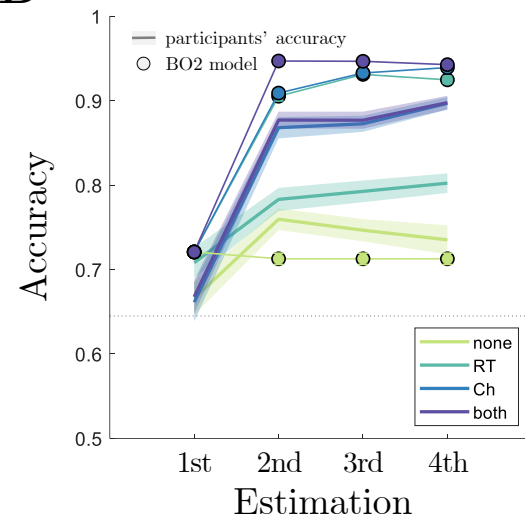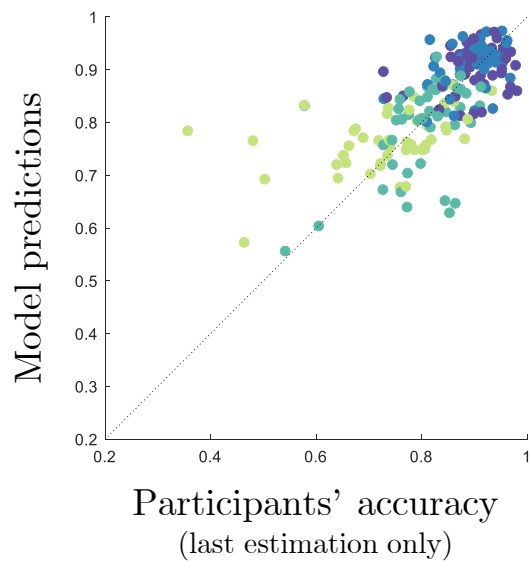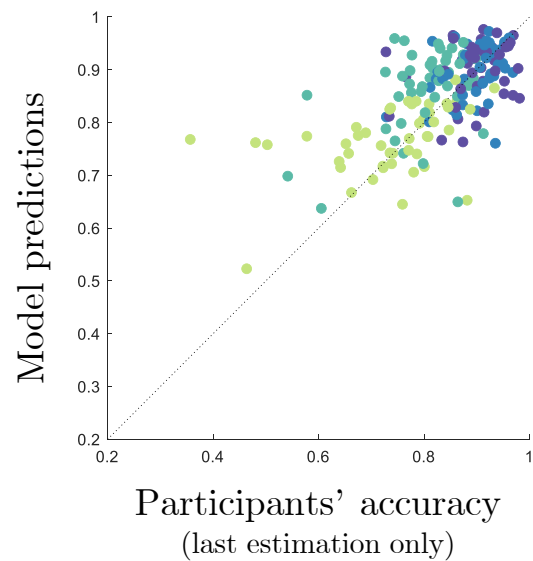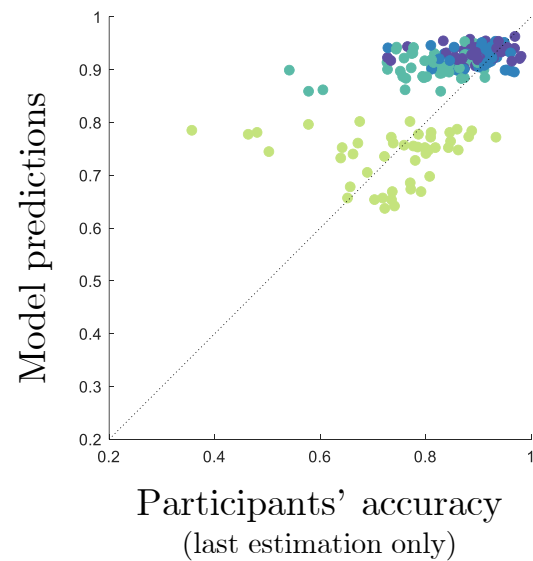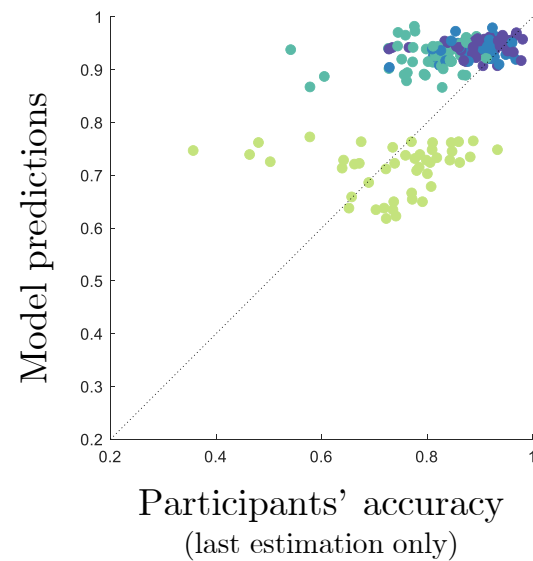

Supplement: S6 Fig — Top: Simulated data (colored dots) superimposed on behavioral data (colored curves) representing the accuracy in the estimation phase for the main RL model (A), the basic RL model (B), the BO model with informative priors (C), and the BO model with uninformative uniform priors (D) in each condition. Shaded areas represent SEM. N = 46. Bottom: accuracy predictions the main RL model (A), the basic RL model (B), the BO model with informative priors (C), and the BO model with uninformative uniform priors (D) as a function a behavioral accuracy in the estimation phase for the last estimation of each participant in each condition. Dashed line represents identity. N = 184. Data and analysis scripts underlying this figure are available at https://github.com/sophiebavard/beyond-choices. (PDF) [file pbio.3002686.s011.pdf]

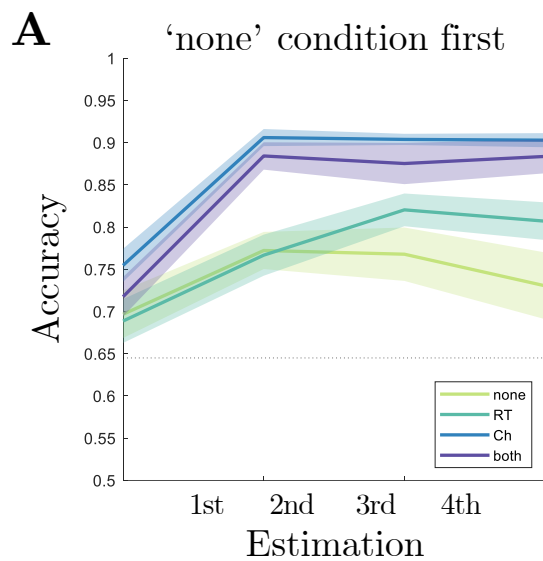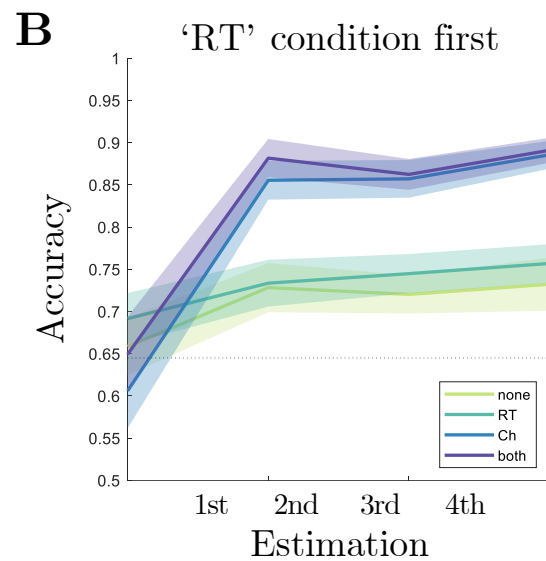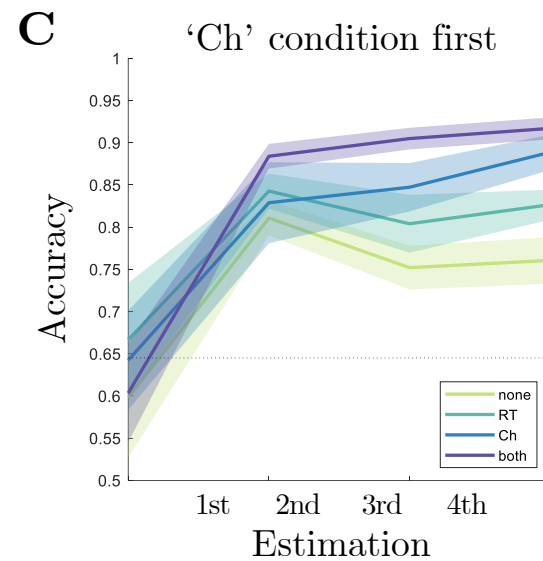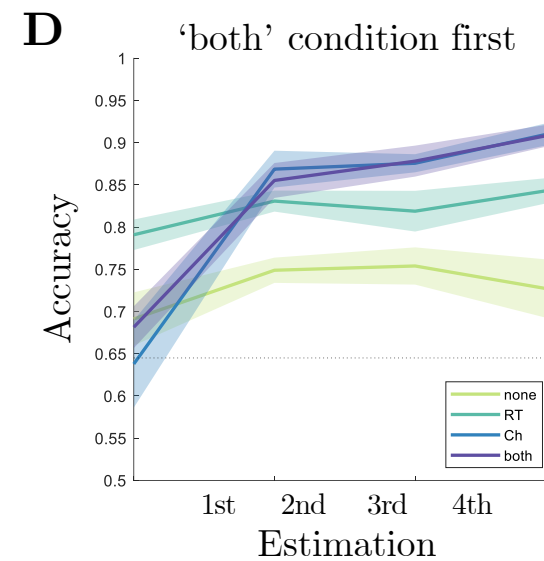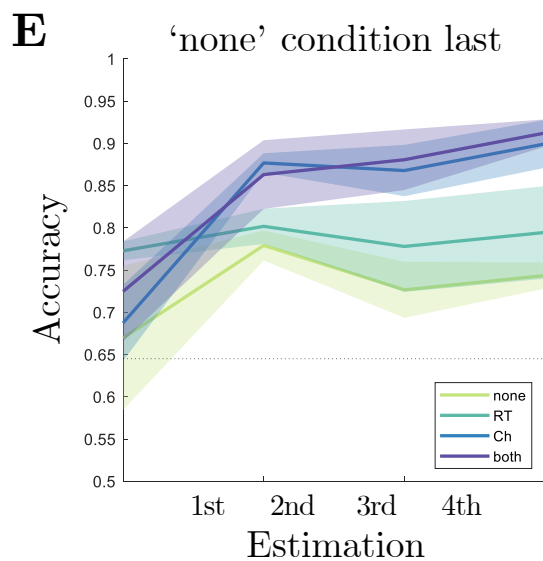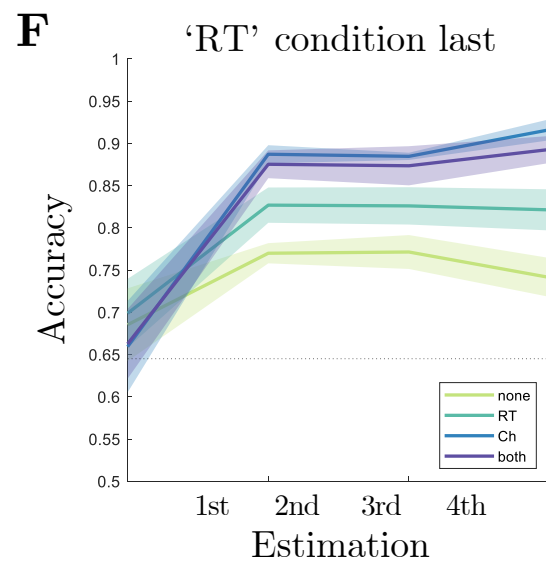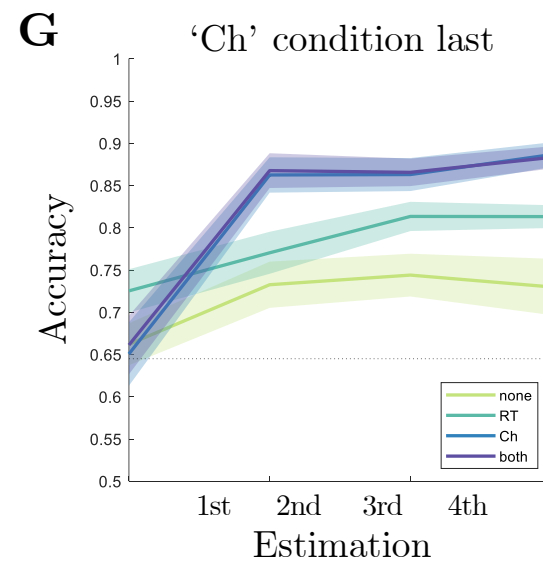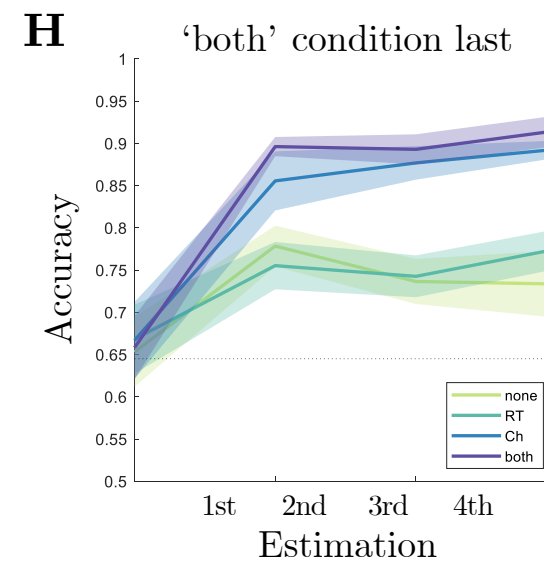

Supplement: S7 Fig — Subset of observers’ accuracy for each estimation as a function of the condition (choice and RT visibility). (A) Observers whose first condition was “none.” (B) Observers whose first condition was “RT.” (C) Observers whose first condition was “Ch.” (D) Observers whose first condition was “both.” (E) Observers whose last condition was “none.” (F) Observers whose last condition was “RT.” (G) Observers whose last condition was “Ch.” (H) Observers whose last condition was “both.” Data and analysis scripts underlying this figure are available at https://github.com/sophiebavard/beyond-choices. (PDF) [file pbio.3002686.s012.pdf]

What is the preferred allocation of this person?  
(please use the mouse)

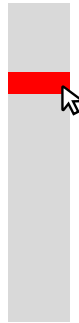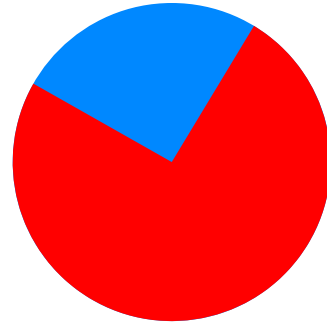

< Continue with space bar >

Supplement: S8 Fig — The figure represents the screen seen by observers to indicate what they thought the dictator’s preference was, by dragging-and-dropping a red (resp. blue for counterbalanced observers) tick on a slider. The < Continue with space bar > line was only displayed after they had made one first click, to avoid perseveration effects. Translated from German for illustration purposes. Data and analysis scripts underlying this figure are available at https://github.com/sophiebavard/beyond-choices. (PDF) [file pbio.3002686.s013.pdf]

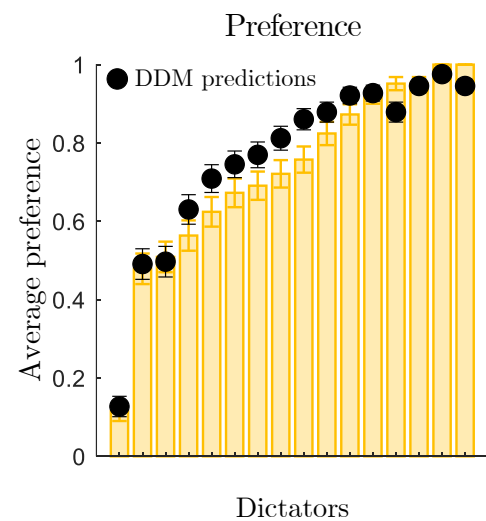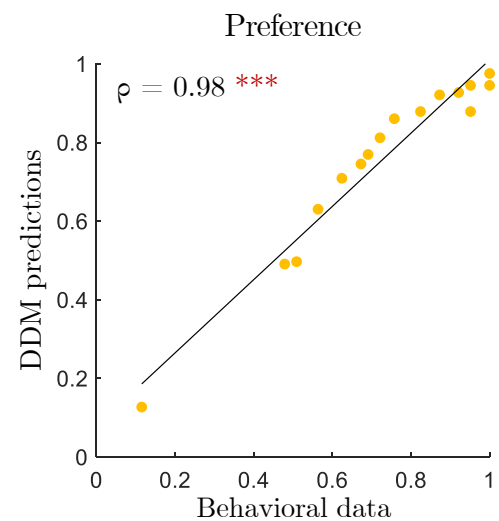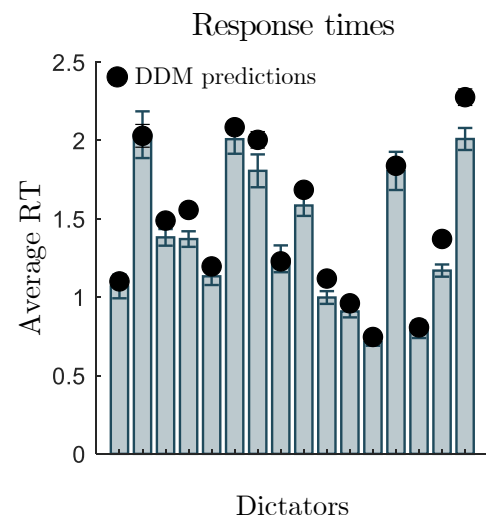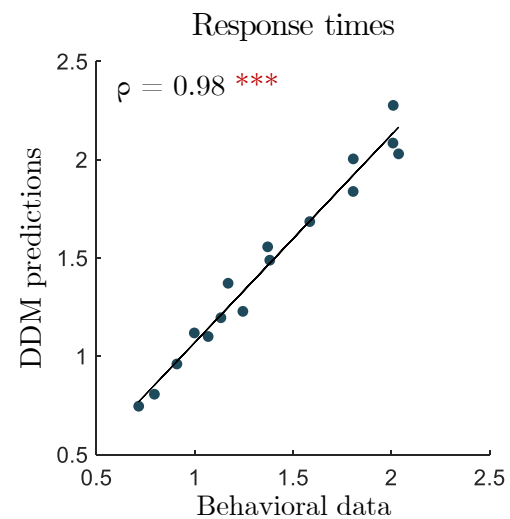

Supplement: S9 Fig — The DDM model was fitted on the Dictator Game data for the 16 dictators, here represented in an increasing order based on their social preference (estimated from their behavioral choices). The DDM is able to match dictators’ behavior both in terms of choices (top panels) and RT (bottom panels). Data and analysis scripts underlying this figure are available at https://github.com/sophiebavard/beyond-choices. (PDF) [file pbio.3002686.s014.pdf]

**A**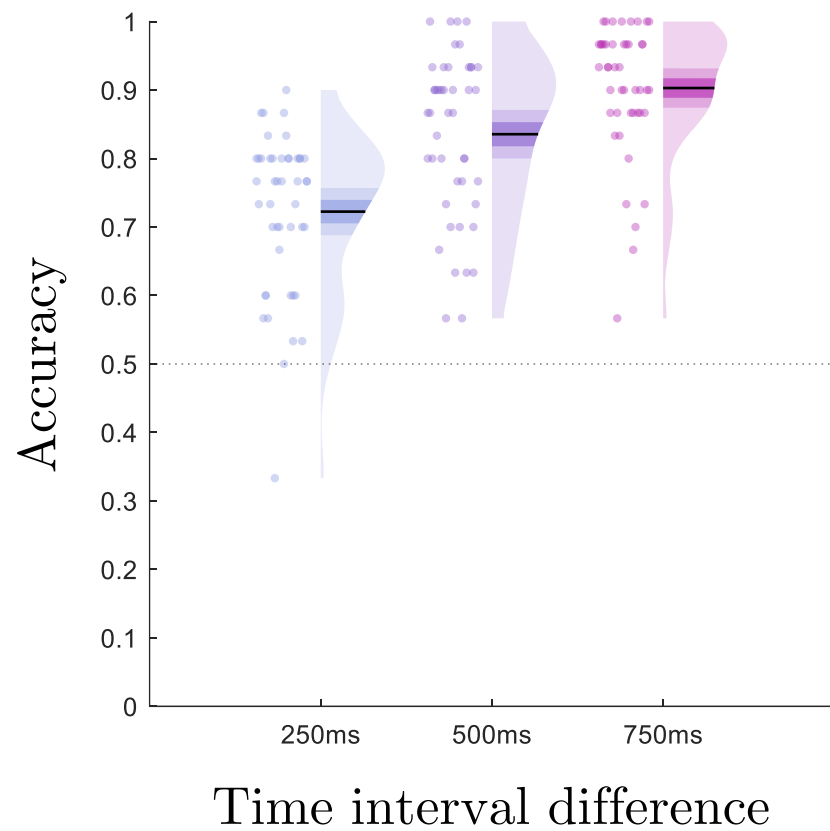**B**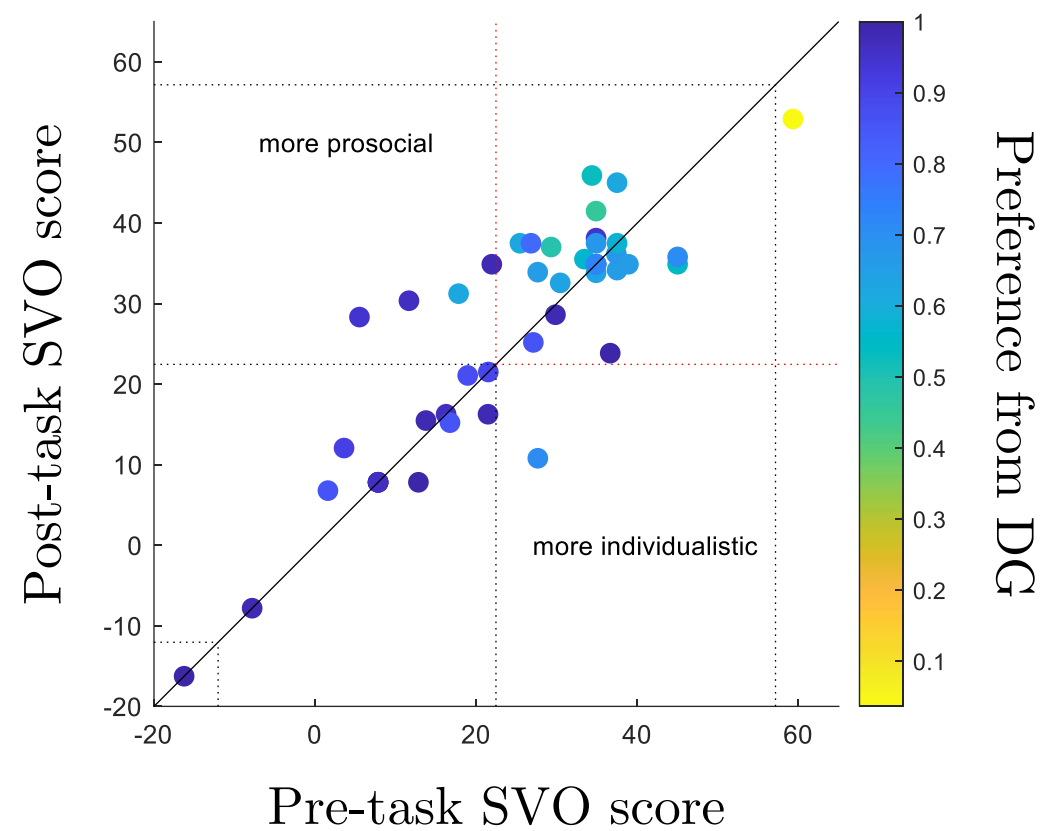

Supplement: S10 Fig — (A) Observers’ accuracy in the time perception task as a function of the difficulty of the trial, i.e., the time interval between 2 stimuli. Points indicate individual average, shaded areas indicate probability density function, 95% confidence interval, and SEM. N = 46. (B) Observers’ post-task SVO score as a function of their pre-task SVO score and their social preference extracted from their choices in the Dictator Game (DG). Black dashed lines represent categorical boundaries: competitiveness/individualism/prosociality/altruism. Red dashed lines represent a change of category from pre- to post-task scores. SVO: Social Value Orientation scale; DG: Dictator Game; N = 46. Data and analysis scripts underlying this figure are available at https://github.com/sophiebavard/beyond-choices. (PDF) [file pbio.3002686.s015.pdf]
