# Supplementary material for: Humans can infer social preferences from decision speed alone
Source: PLoS Biol. 2024 Jun 20;22(6):e3002686. doi: 10.1371/journal.pbio.3002686 (PMC11189591; doi:10.1371/journal.pbio.3002686)
Supplement: S1 Table — The GLMM (generalized linear mixed model with Gamma distribution and identity link function) was fitted on the observers’ RT, with choice visibility in the estimation phase, RT visibility in the estimation phase, and trial duration (i.e., whether the dictator’s RT was short or long), as independent variables. Denotation: Du = duration (fast or slow), Ch = choice visibility (displayed or not), RT = RT visibility (displayed or not), ***p < 0.001. Data and analysis scripts underlying this figure are available at https://github.com/sophiebavard/beyond-choices. (DOCX) [file pbio.3002686.s016.docx]

|  | **Choosing for self** | | | |
| --- | --- | --- | --- | --- |
| **Effect** | **Estimate** | **Std. Error** | ***t*-value** | ***p*-value** |
| Intercept | 1.19 | 0.093 | 12.81 | <.0001 *** |
| Duration | 0.25 | 0.046 | 5.40 | <.0001 *** |
| Choice visibility | -0.031 | 0.033 | -0.94 | .35 |
| RT visibility | 0.015 | 0.033 | 0.45 | .65 |
| Du x Ch | -0.031 | 0.038 | -0.84 | .40 |
| Du x RT | 0.0014 | 0.038 | -0.037 | .97 |
| Ch x RT | 0.0030 | 0.035 | 0.087 | .93 |
| Du x Ch x RT | -0.046 | 0.053 | -0.87 | .38 |
