# Supplementary material for: Humans can infer social preferences from decision speed alone
Source: PLoS Biol. 2024 Jun 20;22(6):e3002686. doi: 10.1371/journal.pbio.3002686 (PMC11189591; doi:10.1371/journal.pbio.3002686)
Supplement: Attachment — Submitted filename: Rebuttal.docx [file pbio.3002686.s017.docx]

In light of the reviews, which you will find at the end of this email, we are pleased to offer you the opportunity to address the comments from the reviewers in a revision that we anticipate should not take you very long. We will then assess your revised manuscript and your response to the reviewers' comments with our Academic Editor. As you will see, one of the comments refers to the inaccessibility of the data repository and Reviewer 3 indicated that they would like to review the data and code for an in-depth review.

We thank the Editor and the Reviewers for the opportunity to revise and re-submit our manuscript, which we believe has been substantially improved with the help of the Reviewers’ feedback. We were delighted to see that all Reviewers were positive about our work, and we were equally happy to integrate their insightful and constructive comments, which have been addressed point-by-point below.

Reviewer #1: Reviewer comments

Summary
The authors investigate the role of response times (RT) in how individuals learn about others' social preferences. The key novel contribution is the finding that RT is predictive of social choice preferences, meaning that observers could infer others' choices were more prosocial versus more selfish when provided only with information about RT (i.e., without information about choices). The authors argue that this deviates from the predictions of an optimal Bayesian model in which people are expected to learn equally well from choices and RT information.

Comments

R1.1. Regarding the Bayesian optimal observer model, something that was unclear in the manuscript is why or in what sense is this model optimal? Does it maximize some performance measure (e.g., accuracy or reward rate per unit time)? A few sentences clarifying this would be beneficial.

We thank the Reviewer for pointing out this lack of clarity. The model is inspired by recent literature on inverted Bayesian inference (Gates et al, 2021; Baker et al, 2017; Jern et al, 2017; Collette et al, 2017; Diaconescu et al, 2014) and seeks to infer the optimal parameters by maximizing the posterior distribution of the parameter set, given all of the evidence collected so far. In other words, the model assumes that observers seek to know what is the most likely set of parameters for this dictator given the observation. We use the framework from Gates et al. (2021): “this follows the use of “rational” in Anderson’s rational analysis framework (Anderson, 1991), which focuses on ideal solutions given assumptions about the problem posed by the environment. Identifying the optimal choice of model (i.e., the model that produces the most accurate inferences about preferences) is roughly equivalent to identifying the most accurate model of choices and RT in preferential choice.” We extended the Introduction and the Methods section as pointed out in our response to the following comments.

R1.2. From the description in the Materials and Methods section, the Bayesian optimal model seems to be comprised of several different models (e.g., Weiner first-passage time, soft-max, WFPT integrated over both choices, and the 'prior equals likelihood' model) which are applied depending on the type of data provided (RT/choices/both/none). The manuscript would benefit from giving a brief description of and motivation for this 'benchmark' model earlier on, such as when the model is first mentioned in the introduction. Doing so would help readers understand the reason for choosing these models as a benchmark for comparison.

We agree with the reviewer’s suggestion and provide this description and motivation of the Bayesian model. As mentioned above, the assumption is that observers infer the dictator’s parameters (including their preference) based on past observations. To make such an inference, observers must have a generative model of the dictator’s decision-making process, which we assumed to be a drift-diffusion model (DDM), which accommodates for both choices and RT. This assumption is supported by a very good fit of the DDM to dictators’ behavior (see the newly added Figure S9 below), as well as the regression and simulations presented in Figure 1D-E. The later results were obtained by implementing the dictators’ decision-making process as a WFPT when both choices and RT were observed (classical DDM), a softmax rule when only choices were displayed, and a WFPT distribution integrated over both choices when only RTs were displayed (note that previous work has shown the equivalence of the DDM and the softmax rule / logit model with regard to choice predictions, e.g., Webb et al, 2020). Therefore, we used the same mechanisms when implementing the Bayesian inference model. We extended the Introduction with a more detailed motivation for using a Bayesian model (lines 95-100), and the Methods section with a better description of the model (lines 775-788):

**Introduction:**
On the other side, recent studies have proposed (inverted) Bayesian inference as the optimal framework underlying the cognitive process of social learning ^24,43–47^, and (quasi-)optimal Bayesian learning has been reported in various fields such as reward-based learning^48,49^ or multi-sensory integration^50^. Motivated by this work, we designed a benchmark Bayes-optimal (BO) model in which the observer’s belief on the dictator’s social preferences and choice processes is updated using Bayes’ rule on prior and current observations.

**Methods:**
*Bayes-optimal model.* Based on prior work suggesting that observers might infer other people’s choice processes via Bayesian inferences^24,43–47^, we tested a model that assumes observers estimate the dictator’s preference using such a Bayesian framework. More specifically, this framework suggests observers infer the optimal parameters by maximizing the posterior distribution of the parameter set, given all the evidence collected so far. In other words, we designed a benchmark Bayes-optimal model which assumes that observers seek to know what is the most likely set of parameters (of a model they assume to be the generative model of the decisions) for this dictator given the observation. To make such an inference, observers must have a generative model of the dictator’s decision-making process (or a model that can be expected to come reasonably close to the true generative model, which itself is unknown). Following previous work^24^, we assumed this model to be a drift-diffusion model (DDM), which indeed provides an excellent account of both choices and RT in our Dictator Game task (**Supp. Figure 9**). In this framework, the drift rate depends on the social preferences and choice options as specified above.

**Supp. Figure 9. DDM simulations on the Dictator Game task.** The DDM model was fitted on the Dictator Game data for the 16 dictators, here represented in an increasing order based on their social preference (estimated from their behavioral choices). The DDM is able to match dictators’ behavior both in terms of choices (top panels) and RT (bottom panels).

We thank the Reviewer for their suggestions, as we believe that these additions significantly improved the manuscript.

R1.3. It is stated in a few places (Abstract/Intro/Discussion) that "from a Bayesian perspective, people should be able to learn equally well from choices and RTs". I think this statement needs a bit more motivation/unpacking as to why choices and RTs should be equally informative when predicting choices. I would think that choice information would be more informative (weighted more heavily when updating a prior) when the task is to infer people's choice preferences. For example, if my task is to infer someone's choice preferences, and I'm given the option to either view their 10 previous choices or 10 previous RTs, I can't really think of a situation in which I'd choose the RT information. If, on the other hand, my task was to infer someone's mean RT, then the RT information would indeed be more informative, and I would disregard the choice information. The reported behavioural results appear to support this strategy: "These results suggest that, in our task, participants used the RT information when no other piece of information was available, but they seemed to disregard RT when the choice information was available" (p.12). Thus, the Bayesian optimal model seems to be somewhat of a 'straw man' rather than a serious competitor against the other models presented in the paper. This could be remedied by providing some further explanation and motivation for this model.

We apologize for the lack of clarity in this regard. What we meant with “to learn equally well from choices and RTs”, is that our analyses of the above-described framework of a Bayesian optimal learner that is able to take either RT, choices, or both into account (depending on which data is available) suggest that such an optimal learner would learn the dictator’s social preferences (almost) as quickly and accurately from RT as compared to choices (see Figure 4D). We agree with the Reviewer’s intuition that for predicting choices and preferred allocations (as in our task), it should be cognitively easier to use choice data rather than RT data, while this might be different when the task is to predict RT. Note, however, that a Bayesian optimal learner would not face any such “cognitive challenge”.

Even though the cognitive plausibility of the Bayesian optimal learner might be questionable, we do not consider it a “straw man”. Note that previous work has used this framework to test predictions of how people learn from observing others’ decisions (Gates et al., 2021; Baker et al., 2017; Jern et al., 2017; Jara-Ettinger et al., 2016; Lucas et al., 2014), and that (quasi-)optimal Bayesian learning performances has been reported in various fields such as reward-based learning (e.g., Behrens et al., 2007; McGuire et al., 2014) or multi-sensory integration (Ernst & Banks, 2002). Therefore, we consider it highly relevant to show that our participants did not reach this optimality, and that our reinforcement-learning-based model provides a more accurate account of their social inference abilities.

Aside from these minor points, I thought the paper was well written and provided a comprehensive analysis of an interesting dataset.

We thank the Reviewer for their positive feedback on the manuscript.

Reviewer #2: The authors examine whether or not people use different combinations of the speed and choices of other peoples decisions when making inferences about social preferences of others. Their main conclusions are the people are able to learn other peoples' social preferences even when they only observed the response time of others'. This is a very interesting finding and, as far as I know, the extent to which response time can be used to infer the social preferences of others has not been carefully examined, which makes this study a novel and exciting addition to the literature.

The manuscript is short and sweet -- they use a direct and straightforward design and show a clear main effect of response time in the accuracy of the inferences drawn about others' social preferences. They also use the response time to update a simple reinforcement learning model to allow the model to learn to predict subsequent choice and response times. The model is already fairly established in other applications, so simply adding response time in the current context is a natural step. The authors also use a Bayesian optimal model as a point of comparison and evaluating their RL model. Although I didn't examine this BO model closely, the authors show rather large qualitative (and quantitative) differences in the accuracy of the two models, with the RL model clearly outperforming the BO model.

Given the straightforward approach (in terms of the experimental design and computational modeling) as well as the very clear experimental and computational modeling results, I really don't have much to offer for suggested improvements. The authors provided a very accessible manuscript and have cited all the relevant literature that I can think of, so I would recommend acceptance.

We thank the Reviewer for their interest in our work and positive comments on the manuscript.

Reviewer #3: The manuscript addresses an experiment in which participants observe information about others' decisions or response times to make decisions in the context of prosocial behavior, and finds that people can infer others' preferences from the process by which they make decisions. There is much to like about the paper, and although the details of the developed learning model are outside of my area of expertise to assess, I find myself to be enthusiastic overall.

R3.1. However, although it appears that there is documentation of the materials, data and code available on github, the link provided lead me to a 404 error message. Regarding the OSF project related to the preregistration, I believe I did not have reading rights. Therefore, it was not possible for me to review the paper in depth. I would be happy to do so in another round of reviews. I did find and review the preregistration, which clearly stipulated the manipulated conditions and hypotheses but gave only sparse indications of the overall procedure or analyses to be expected. In the manuscript, it would be great if it was even easier to spot which results related to preregistered hypotheses. In any case, I find it highly commendable that the authors provided a preregistration.

As the Reviewer correctly points out, we planned to make the Github repository public upon publication of our study. We have now uploaded all data and relevant scripts to the (now public) repository, which the Reviewer can access at <https://github.com/sophiebavard/beyond-choices>. Regarding the OSF project, we only used the platform for the pre-registration, so the project was also kept private; we have now corrected this as well and the Reviewer should be able to access both the preregistration at <https://osf.io/tz4dq> and the project at <https://osf.io/fseyw/>. Following the Reviewer’s recommendation, we improved the Results section and the Supplementary Materials by pointing out when the reported results related to preregistered hypotheses. We thank the Reviewer for this suggestion and their interest in reviewing our data, and we are looking forward to reading their feedback.

In addition, I have a few comments about the behavioral results that I believe should be addressed in a revision of the manuscript.

Learning about Dictators' Preferences (Estimation Phase)

R3.2. In the results section (p. 5, bottom paragraph), the authors report that observers' learning of the dictators' preferences was better than chance overall, and separately for the four conditions induced (RT only, choice only, both, none), in correspondence with the preregistered hypothesis that "(1) the mean accuracy of participants to predict the dictators' social preference will be higher than chance in three out of four conditions, [and] (2) this accuracy will increase with the amount of information provided to the participants". The paragraph is quite densely written, so that perhaps it might be helpful to add a bit of explanation back in. For instance: starting with line 178, do you report first the overall effect of learning (in all conditions), then the effect separately for the RT only condition, then a post-hoc contrast between the choice only and both conditions, and then the effect separately for the none condition? Perhaps a table would help summarize these results efficiently, and give you more space for elaborating in the text.

We agree with the Reviewer that the paragraph was somewhat convoluted. Following the Reviewer’s suggestion, we reframed the paragraph as follows (lines 181-195), and we added a table summing up all (Bonferroni corrected) post-hoc t-tests:

Then, according to our main preregistered hypothesis, we analyzed observers’ accuracy in estimating the dictators’ preference (note that for readability, statistical tests of this paragraph are summarized in **Table 1** rather than reported in the text). On average, observers were able to learn above the empirical chance level (see **Methods**, *t*(45)=22.59, *p*<.0001, *d*=3.33), even in the ‘RT only’ condition (**Figure 2A**). Surprisingly, observers’ accuracy was above the empirical chance level in the ‘none’ condition as well (**Figure 2A**). However, the correlation between the dictators’ true preference and the observers’ last estimation was not significant in this condition (Spearman’s *ρ*(182)=0.13, *p*=.071), whereas it was significant in all conditions where some information was provided (RT only: Spearman’s *ρ*(182)=0.41, *p*<.0001; choice only: Spearman’s *ρ*(182)=0.84, *p*<.0001; both: Spearman’s *ρ*(182)=0.84, *p*<.0001). This suggests that observers learned to distinguish more prosocial from more selfish dictators in conditions with information but not in the ‘none’ condition, where they mostly used their own preference (**Figure 2B**, **Supp. Figure 3**). Furthermore, while accuracy was higher in the ‘both’ condition than in the ‘RT only’ condition, observers seemed to learn equally well in the ‘choice only’ and ‘both’ conditions (**Figure 2A**). The latter result was in contrast with our predictions. All statistical analyses across conditions are reported in **Table 1**.

**Table 1. Pairwise comparisons of average accuracy per condition.** Emp: empirical, ***p*<.01, ****p*<.001, Bonferroni-corrected for between-conditions pairwise comparisons.

R3.3. Moreover, I expected to see an interaction effect reported about the effect of condition x time of measurement (before first trial, after first trial, after second trial, etc.) on the perception of the dictators' preferences. It seems to me like the perceptions of the dictators' preferences would probably get better across the four trials, maybe particularly so in the both condition and perhaps not at all in the none condition.

Thank you for pointing this out, we believe that reporting this interaction would be very relevant early on in this part of the Results section, which we modified accordingly (lines 195-202):

Finally, to get a more fine-grained understanding of learning dynamics, we amended the preregistered analysis and performed a 2x4 ANOVA with factors condition (‘none’, ‘RT only’, ‘choice only’, ‘both’) x estimation number (1^st^, 2^nd^, 3^rd^, 4^th^). Consistent with our results so far, we found significant main effects of both conditions (*F*(3,135) = 36.84, *p* < .0001, *η²* = .45, Huynh–Feldt corrected) and estimation number (*F*(3,135) = 80.71, *p* < .0001, *η²* = .64, Huynh–Feldt corrected), and more interestingly a significant interaction (*F*(9,405) = 13.58, *p* < .0001, *η²* = .23, Huynh–Feldt corrected), suggesting that observers learned faster in the ‘choice only’ and ‘both’ conditions (**Figure 2A**).

R3.4. Regarding the dictators' preference in the methods section, it didn't quite become clear to me how the participants indicated what they thought the dictators' preferences were.

Thank you for pointing this out, we added a short description in the Methods section (lines 588-590):

To do so, they were asked to move a tick on a slider, which simultaneously changed the visual proportion of an allocation displayed next to the slider (**Supp. Figure 8**).

**Supp. Figure 8. Visual representation of the estimation phase.** The figure represents the screen seen by observers to indicate what they thought the dictator’s preference was, by dragging-and-dropping a red (resp. blue for counterbalanced observers) tick on a slider. The < Continue with space bar > line was only displayed after they had made one first click, to avoid perseveration effects. Translated from German for illustration purposes.

Extrapolation to Unseen Decisions

R3.5. Minor point: I noticed that the terminology and the perspective on the data changed a bit in this section. Where you referred to RT only condition before, you now refer to the RT visibility (which presumably also addressed the both condition). For consistency and to increase the ease with which readers can follow the presented work, perhaps it might be good to keep the perspective on the data constant throughout, or to alert the readers to the difference?

We agree with the Reviewer and pointed at the new terminology in the relevant paragraph (lines 218-221):

From here on, in contrast with ‘choice only’ and ‘RT only’ conditions, we define the ‘choice visibility’ and ‘RT visibility’ as orthogonal factors in our design representing whether or not the choice (resp. RT) information was displayed in each condition; for example, choice visibility is set to 1 in the ‘choice only’ and ‘both’ conditions, and to 0 in the ‘RT only’ and ‘none’ conditions.
