# Supplementary material for: Humans can infer social preferences from decision speed alone
Source: PLoS Biol. 2024 Jun 20;22(6):e3002686. doi: 10.1371/journal.pbio.3002686 (PMC11189591; doi:10.1371/journal.pbio.3002686)
Supplement: Attachment — Submitted filename: Rebuttal2.docx [file pbio.3002686.s018.docx]

Reviewer #1 (Russell J. Boag): The authors have addressed my comments satisfactorily.

We thank Dr. Boag for the insightful comments that critically improved our paper and the positive evaluation of the revised manuscript.

Reviewer #3: To the degree that the manuscript coincides with my expertise, I found most of my comments addressed appropriately. However, I continue to quarrel with details relating to the Open practices for this manuscript.

We thank the Reviewer for the insightful comments that critically improved our paper, and for allowing us to improve our Open practices.

Unfortunately, the linked OSF project was empty. Therefore, performing a review based on the data and materials that should presumably be contained therein was not possible. I think it would be great if materials, such as the program used to elicit responses from participants, were made available in this repository. Given that PLOS Biology is an outlet that is committed to transparency regarding research protocols, this seems like an important step to me.

We thank the Reviewer for allowing us to clarify this point. The OSF registry we linked in the Methods section of the manuscript (https://osf.io/tz4dq) redirects to the preregistration document, not the OSF project. We only used OSF as a means to publish the preregistration, but all of the data and code is openly accessible on GitHub. For maintenance reasons, it appears safer to us to only store the materials in a single place (GitHub) and avoid duplicates. We thank the Reviewer for raising this potential confusion, and to avoid confusion of the readers, we have added a description to the OSF project (https://osf.io/fseyw/) which redirects to the GitHub repository.

In the github project, I could not locate a file that looked like it contained materials, too. I assume the data might be stored in .mat files (e.g., data_fig.mat), which makes them inaccessible to users like me who are not running Matlab. Might I suggest saving a more accessible version of the raw data, in line with the FAIR principle of interoperability?

We thank the Reviewer for this suggestion. We have added all datasets in a .csv format in a dedicated folder in the GitHub repository (https://github.com/sophiebavard/beyond-choices), which we hope to be a more accessible version of the data. We have also updated the Readme file to make this addition explicit, as well as to add a link redirecting to the OSF preregistration document.
